# Efficacy and Safety of Hormone Replacement Combined With Escitalopram in the Treatment of Chronic Insomnia in Perimenopausal Women: A Randomized Controlled Trial

**DOI:** 10.1111/cns.70470

**Published:** 2025-06-12

**Authors:** Hui Chen, Shufang Wu, Hongbin Chen, Guiying Zeng, Weiwei Wu, Xinyan Chen, Xiujuan Chen, Ronghua Chen, Yingchun Xiao

**Affiliations:** ^1^ Department of Gynecology, Fujian Maternity and Child Health Hospital Affiliated Hospital of Fujian Medical University Fuzhou China; ^2^ Department of Neurology Fujian Medical University Union Hospital Fuzhou China; ^3^ Fujian Key Laboratory of Molecular Neurology Fujian Medical University Fuzhou China; ^4^ Institute of Clinical Neurology Fujian Medical University Fuzhou China

**Keywords:** chronic insomnia, diagnostic indicators, escitalopram, hormone replacement therapy, perimenopausal women

## Abstract

**Aims:**

To explore the efficacy of Femoston plus escitalopram for perimenopausal women with chronic insomnia and the relevant biomarkers.

**Methods:**

A total of 166 patients randomly received: escitalopram plus placebo (Escitalopram Group), Femoston plus placebo (Hormone Group), and Femoston plus escitalopram (Combined Group) for 3 months and followed for 2, 4, 8, and 12 weeks. The primary efficacy endpoint was changes in Pittsburgh Sleep Quality Index Scale (PSQI), Insomnia Severity Index Scale (ISI), and Epworth Sleepiness Scale (ESS) scores at week 12 from baseline. Secondary endpoints included changes in the Modified Kupperman Menopausal Index Scale (KMI) scores, blood 5‐HT neurotransmitters and their receptor, and blood sex hormone levels during the treatment.

**Results:**

Compared with baseline levels, all groups displayed increased serum 5‐HT levels and decreased serum FSH levels, with more significant changes in the combined group. Compared with the other two groups, the combined group reported a gradual increase in serum E2 levels and a gradual decrease in serum LH levels, and the lowest KMI, ESS, ISI, and PSQI scores at weeks 4 and 12. The PSQI score was negatively correlated with serum 5‐HT and E2 and positively correlated with serum FSH and LH levels, respectively.

**Conclusion:**

Femoston plus escitalopram improves chronic insomnia in perimenopausal women. Serum levels of 5‐HT, E2, FSH, and LH may objectively indicate the clinical severity of chronic insomnia in this population.

## Graphical abstract

Compared with hormone or escitalopram alone, a combined therapy of Femoston with escitalopram may be more effective in the treatment of perimenopausal chronic insomnia. Serum levels of 5‐HT, E2, FSH, and LH may serve as objective indicators to assess the disease severity in perimenopausal women.

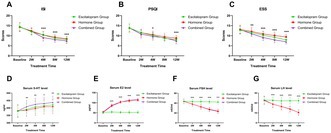

## 1 Introduction

Perimenopause refers to a period from the menstrual cycle and ovarian dysfunction, near menopause, to the last menstrual period within 1 year [1]. Perimenopausal women may experience a series of clinical symptoms, including insomnia, hot flashes, night sweats, menstrual disorders, anxiety, depression, etc. [2] Of all these symptoms, chronic insomnia is one of the main symptoms. These manifestations can seriously impact family harmony and the quality of life of perimenopausal women [3]. Epidemiological findings in both national and international studies have shown that about 40%–60% of perimenopausal women may be susceptible to this sleep disorder [4, 5].

To date, the pathogenesis of perimenopausal chronic insomnia remains obscure. Several hypotheses have been proposed to account for the mechanisms of chronic insomnia in perimenopause, including changes in sex hormone levels, frequent vasodilatory symptoms, and changes in the levels of neurotransmitters, such as 5‐hydroxytryptamine (5‐HT) and its receptors [6, 7, 8, 9, 10]. In addition, evidences show that estrogen can regulate sleep by influencing the synthesis and re‐uptake of 5‐HT to regulate the 5‐HT pathway [9, 11, 12]. These findings suggest that estradiol (E2), follicle‐stimulating hormone (FSH), luteinizing hormone (LH), and 5‐HT may play an important role in the development of chronic insomnia in perimenopausal women.

Chronic insomnia is commonly assessed by subjective sleep scales, the accuracy of which is often compromised due to variations. Therefore, objective indicators are urgently needed to determine the severity of chronic insomnia in perimenopausal women. Studies have shown that perimenopausal women have low blood levels of E2 and 5‐HT and high levels of FSH and LH [13, 14]. Platelets can serve as peripheral models in studying serotonergic neuronal activity, and the mechanisms of 5‐HT uptake and release in platelets are similar to those in the central nervous system [15, 16, 17]. It is intriguing to investigate whether levels of serum sex hormones, platelet 5‐HT, and their receptor can reflect the severity of chronic insomnia in perimenopausal women.

To address chronic insomnia in perimenopausal women, various estrogen therapeutic schemes, such as Femoston, have been undertaken [18]. However, this therapy is slow‐acting, and relapses may occur after drug discontinuation [19]. Available studies have demonstrated that selective serotonin reuptake inhibitors (SSRIs), such as escitalopram, can somewhat improve chronic insomnia and subjective sleep quality in perimenopausal women [20, 21]. However, escitalopram alone is ineffective in alleviating vasomotor disturbances in perimenopausal women [22].

Compared with escitalopram alone, a combination of estrogen and escitalopram has been found to produce a faster action onset and better efficacy in treating depression and vasomotor symptoms in perimenopausal women [23]. However, it remains unresolved whether such a combination may be beneficial to perimenopausal women with chronic insomnia. Therefore, a randomized controlled trial was conducted in which we hypothesized that Femoston plus escitalopram may have better efficacy for perimenopausal women with chronic insomnia. The findings of this study may provide clinical insights into the treatment of perimenopausal chronic insomnia.

## 2 Materials and Methods

### 2.1 Research Participants

This study included patients with perimenopausal chronic insomnia who visited the outpatient and inpatient departments of Fujian Medical University Union Hospital and Fujian Maternity and Child Health Hospital from October 2021 to December 2022.

Inclusion criteria were as follows: (1) changes in the length of two adjacent menstrual cycles ≥ 7 days within a continuous 10‐month period or a menstrual pause ≥ 60 days between menstrual periods, with the last menstruation as the end point of the menopausal transition and 1 year after menopause as the end point of perimenopause; (2) intact ovaries and uterus; (3) no history of chemotherapy and/or radiotherapy; (4) no medications for adverse perimenopausal symptoms in the past 3 months; (5) meeting the diagnostic criteria for chronic insomnia in the International Classification of Sleep Disorders, 3rd edition (ICSD‐3) [24], with sleep difficulties and related daytime symptoms occurring at least 3 times a week and lasting for at least 3 months; (6) capability to complete the questionnaire independently, sign informed consent, and voluntarily join the study.

The exclusion criteria included any of the following: (1) evidence of neurological diseases, mental illness, and major organic diseases; (2) a surgical history in the past 6 months or a previous surgery for the uterus and bilateral appendages; (3) communicative incompetence due to hearing, intellectual, and language impairments; (4) ongoing treatments related to sleep disorders; (5) people with severe diabetic complications or insulin use; (6) people with an outpatient blood pressure equivalent to grade 3 hypertension (systolic blood pressure ≥ 180 mmHg and/or diastolic blood pressure ≥ 110 mmHg).

### 2.2 Grouping and Treatment

This study was approved by the Ethics Committee of Fujian Maternity and Child Health Hospital (Ethics Batch No. 2021KLRD09018). Of the total 206 perimenopausal patients with chronic insomnia recruited, 180 patients were finally enrolled according to the inclusion and exclusion criteria.

Patients were numbered according to the enrollment sequence and randomly grouped at a 1:1:1 allocation ratio according to the order of random numbers generated with SPSS 17.0 software (SPSS Inc., Chicago, IL, USA). An independent researcher used the algorithm to perform rater masking. The randomized information of each eligible patient was sealed in an opaque envelope that corresponded to the patient enrollment number.

Participants were randomized into three 12‐week treatment regimens: escitalopram + placebo (Escitalopram group), Femoston + placebo (Hormone group), and Femoston plus escitalopram (Combined group). In the Escitalopram group, escitalopram (Bailot, Sichuan Kelun Pharmaceutical Co. Ltd.) was administered at a dosage of 10 mg once daily; in case of adverse events (AEs) after the administration, the dose was reduced to 5 mg once daily for 3 days and increased to 10 mg for maintenance therapy if the AEs were relieved; the placebo was a Femoston analogue and was administered in the same dosage regimen as Femoston. In the Hormone group, Femoston 1/10 (Solvay Pharmaceutical Co. Ltd.), which consists of a white tablet (estradiol 1 mg) and a gray tablet (estradiol 1 mg + dydrogesterone 10 mg), was given according to the following regimen: 1 tablet orally every day on a 28‐day treatment course, with the white tablet taken on the first 14 days and the gray tablet on the next 14 days and a second course starting from day 29. If the discomfort associated with estrogen insufficiency did not improve, the patients received Femoston 2/10 (Solvay Pharmaceuticals Ltd.), composed of a brick red tablet (estradiol 2 mg) and a yellow tablet (estradiol 2 mg + dydrogesterone 10 mg), and observed the same dosage scheme. The placebo was an escitalopram analogue, given at a dosage of 10 mg once daily. In the Combined group, Femoston and escitalopram were taken simultaneously according to the above schemes.

During the treatments, contraindicated drugs were as follows: triptans, antipsychotics, catecholamines, glucocorticosteroids, monoamine oxidase inhibitors, and β‐receptor blockers. The use of the following drugs was allowed: antiplatelet agents, anticoagulants, B vitamins, angiotensin‐converting enzyme inhibitors, angiotensin receptor blockers, and calcium channel antagonists.

### 2.3 Baseline Characteristics

Baseline data were collected, including age, education, body mass index (BMI), KMI score, ISI score, PSQI score, ESS score, and platelet 5‐HT, platelet 5‐HT7R, serum E2, FSH, LH, and 5‐HT levels. The aforementioned scale evaluation and blood tests were performed at weeks 2, 4, 8, and 12 after the treatment.

### 2.4 Outcome Measurements

The primary efficacy endpoint was changes in ISI, PSQI, and ESS scores at week 12 from baseline. Secondary endpoints included changes in KMI scores, blood 5‐HT neurotransmitters and their receptor, and blood sex hormone levels during the 12‐week treatment.

### 2.5 Collection and Testing of Blood Samples

Fasting blood specimens were collected before the treatment and at weeks 2, 4, 8, and 12 after the treatment. Platelet 5‐HT, 5‐HT7R, and serum 5‐HT, E2, FSH, and LH levels were measured by enzyme‐linked immunosorbent assay. A detailed description of these analytical methods is given in the Supporting Information.

### 2.6 Follow‐Up and Evaluation of Adverse Events

The follow‐up of this study lasted for 12 weeks. KMI, ISI, PSQI, and ESS scales were adopted to evaluate the therapeutic effect at weeks 2, 4, 8, and 12 after the treatment. Blood pressure, blood routine index, liver function, renal function, electrolytes, and electrocardiogram were monitored during the treatment. The severity of AEs was assessed by the Treatment Emergency Symptom Scale (TESS) [25].

### 2.7 Statistical Analysis

Statistical analyses were performed with Statistical Product and Service Solutions (SPSS) 20.0 software. Primary efficacy was analyzed with a per‐protocol set (PPS), which included all individuals who completed the study according to that protocol. Data were checked for normality using frequency histograms, Q‐Q plots, and the Kolmogorov–Smirnov test. The measurement data were expressed as mean ± standard deviation (*x* ± SD). Differences in baseline data between groups were compared using one‐way ANOVA or the Kruskal‐Wallis H test. Count data were expressed in frequency or percentage (%) and the inter‐group comparisons were analyzed by chi‐square tests or Fisher exact tests. Between‐ and within‐group comparisons of variables at weeks 2, 4, 8, and 12 were assessed by Generalized Estimation Equations (GEEs). The correlations of peripheral blood indices with KMI, PSQI, ESS, and ISI scores were analyzed by the Pearson partial correlation test. A bilateral *p* value of < 0.05 was considered statistically significant.

## 3 Results

### 3.1 Comparison of Baseline Data

**FIGURE 1:**
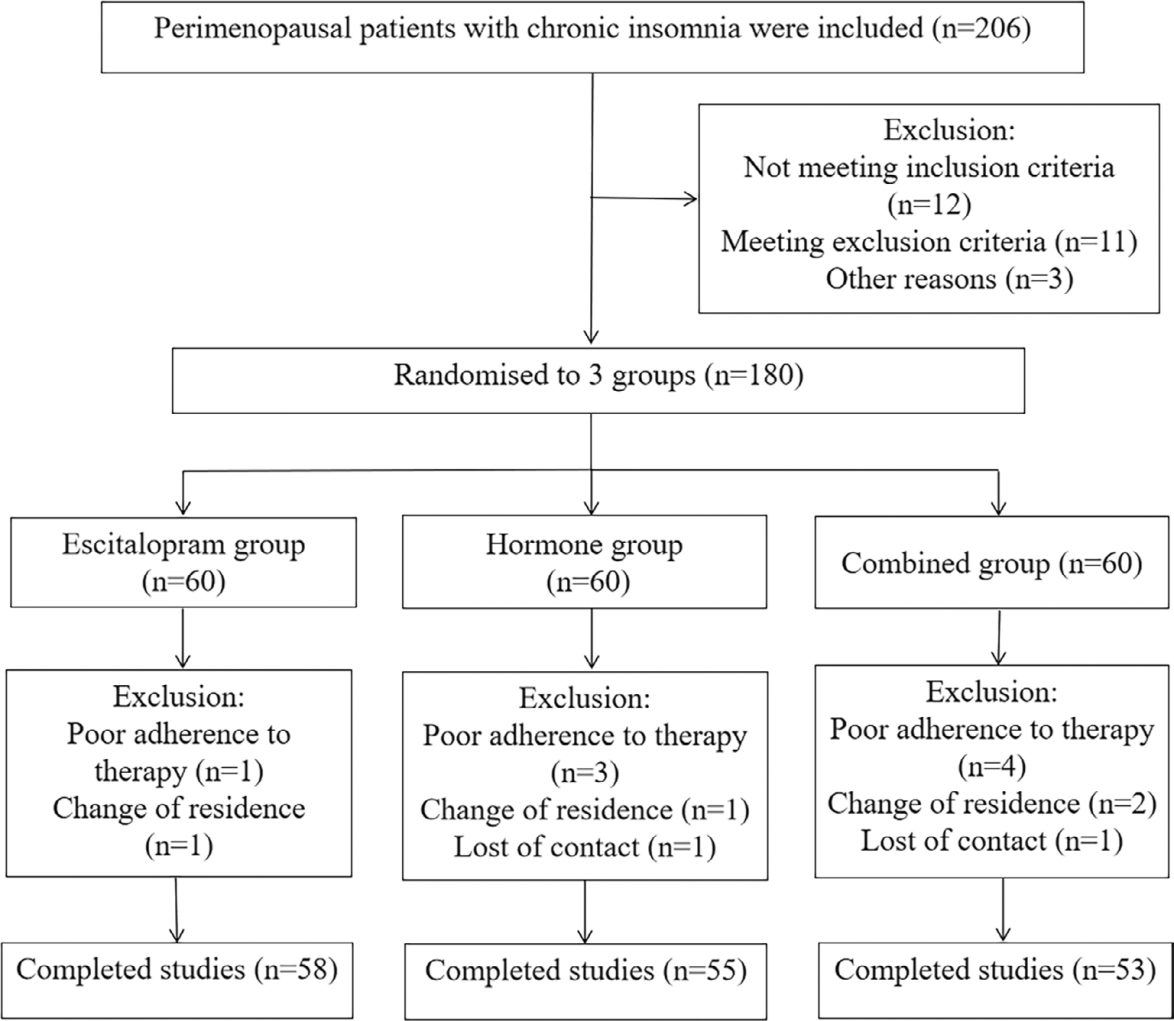
Flowchart of patient enrollment.

Of the total 206 perimenopausal patients with chronic insomnia recruited in this study (Figure 1), 26 patients were excluded, and the remaining 180 patients were randomly divided into three groups of 60 patients each. A total of 166 patients finally completed the 12‐week study (58 patients in the Escitalopram group, 55 in the Hormone group, and 53 in the Combined group). The baseline characteristics of the enrolled participants did not differ significantly between the three groups (Table 1).

**TABLE 1:** Comparison of baseline data for the three groups.

Item	Escitalopram group (*n* = 58)	Hormone group (*n* = 55)	Combined group (*n* = 53)	*p*
Age, years	49.69 ± 2.95	49.45 ± 2.18	49.32 ± 2.42	0.741
Education years, year	7.14 ± 4.82	6.95 ± 3.82	7.28 ± 3.91	0.917
BMI, kg/m^2^	22.29 ± 2.49	22.28 ± 1.93	22.05 ± 2.16	0.813
KMI Score	28.14 ± 4.18	28.04 ± 4.24	28.64 ± 4.16	0.725
ISI Score	14.47 ± 2.01	14.31 ± 2.09	14.30 ± 2.18	0.895
PSQI Score	13.88 ± 1.93	13.82 ± 2.04	13.89 ± 1.95	0.980
ESS Score	12.78 ± 1.94	13.13 ± 2.15	12.98 ± 1.83	0.638
Platelet 5‐HT, ng/mL	428.74 ± 53.50	422.11 ± 68.45	426.74 ± 60.04	0.840
Serum 5‐HT, ng/mL	377.91 ± 39.96	380.26 ± 39.46	379.36 ± 42.32	0.953
Serum E2, pg/mL	52.11 ± 6.38	53.60 ± 5.43	52.60 ± 5.56	0.386
Serum FSH, mIU/mL	43.57 ± 4.86	43.49 ± 4.94	43.40 ± 4.62	0.983
Serum LH, mIU/mL	22.90 ± 4.01	22.49 ± 4.18	22.88 ± 3.54	0.829
5‐HT7R, ng/L	671.53 ± 75.08	666.28 ± 77.45	660.82 ± 60.97	0.735

*Note:* Data are shown as mean ± SD. Abbreviations: 5‐HT, 5‐hydroxytryptamine; 5‐HT7R, 5‐hydroxytryptamine 7 receptor; E2, Estradiol; ESS, Epworth Sleepiness Scale; FSH, Follicle‐stimulating Hormone; ISI, Insomnia Severity Index Scale; KMI, Modified Kupperman Menopausal Index; LH, Luteinizing Hormone; PSQI, Pittsburgh Sleep Quality Index Scale.

### 3.2 Score Changes of Each Scale

The changes in KMI scores in the three groups are shown in Table S1 and Figure S1. Compared with those before the treatment, all three groups reported a marked decrease in KMI scores at weeks 2, 4, 8, and 12 after the treatment, indicating a gradual downward trend, with the most significant changes in the combined group.

**FIGURE 2:**
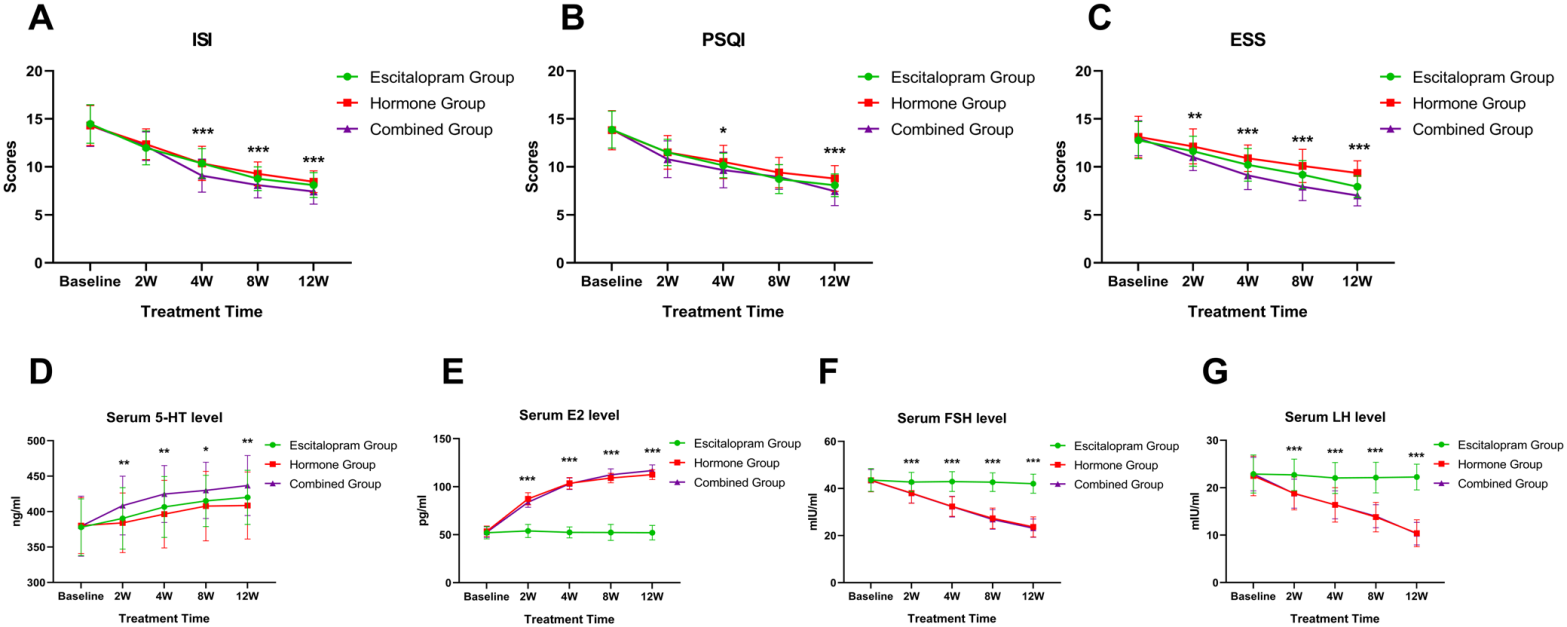
Changes in subjective sleepiness scale scores and serum 5‐HT and sex hormone levels at different time points in the three groups. ISI, Insomnia Severity Index Scale; PSQI, Pittsburgh Sleep Quality Index Scale; ESS, Epworth Sleepiness Scale; 5‐HT, 5‐hydroxytryptamine; E2, Estradiol; FSH, Follicle‐stimulating Hormone; LH, Luteinizing Hormone. Comparison of three groups at the same time point: *p < 0.05, **p < 0.01, ***p < 0.001.

The subjective sleep status of patients was evaluated by ISI, PSQI, and ESS scales (Table 2 and Figure 2). Compared with those before the treatment, the ISI, PSQI, and ESS scores decreased in all three groups after the treatment. At week 2, no significant difference in ISI scores was evident across the three groups, while PSQI and ESS scores were significantly lower in the combined group than in the other two groups. At weeks 4, 8, and 12, ISI and ESS scores were noticeably lower in the combined group than in the other two groups. After the treatment, compared with those in the other two groups, PSQI scores in the combined group were not significantly different at week 8 but markedly lower at week 12.

**TABLE 2:** Changes in subjective sleep scale scores and serum 5‐HT and sex hormone levels in the three groups before and after the treatment.

Item	Time	Escitalopram group (*n* = 58)	Hormone group (*n* = 55)	Combined group (*n* = 53)	*p*_a_	*p*_b_	*p*_c_
ISI Score	0 W	14.47 ± 2.01	14.31 ± 2.09	14.30 ± 2.18	0.683	0.986	0.679
	2 W	11.97 ± 1.77***	12.36 ± 1.60***	12.15 ± 1.49***	0.205	0.470	0.545
	4 W	10.36 ± 1.54***	10.36 ± 1.78***	9.08 ± 1.73***	0.996	< 0.001	< 0.001
	8 W	8.76 ± 1.23***	9.27 ± 1.24***	8.09 ± 1.33***	0.026	< 0.001	0.006
	12 W	8.09 ± 1.30***	8.44 ± 1.14***	7.43 ± 1.32***	0.124	< 0.001	0.008
PSQI Score	0 W	13.88 ± 1.93	13.82 ± 2.04	13.89 ± 1.95	0.869	0.857	0.984
	2 W	11.50 ± 1.37***	11.51 ± 1.75***	10.79 ± 1.91***	0.975	0.040	0.024
	4 W	10.14 ± 1.29***	10.51 ± 1.73***	9.66 ± 1.86***	0.194	0.013	0.116
	8 W	8.72 ± 1.51***	9.40 ± 1.57***	8.94 ± 1.28***	0.019	0.094	0.403
	12 W	8.07 ± 1.18***	8.78 ± 1.34***	7.45 ± 1.49***	0.003	< 0.001	0.015
ESS Score	0 W	12.78 ± 1.94	13.13 ± 2.15	12.98 ± 1.83	0.358	0.701	0.563
	2 W	11.62 ± 1.57***	12.13 ± 1.83***	11.00 ± 1.40***	0.111	< 0.001	0.026
	4 W	10.21 ± 1.70***	10.89 ± 1.38***	9.11 ± 1.48***	0.018	< 0.001	< 0.001
	8 W	9.17 ± 1.47***	10.09 ± 1.74***	7.92 ± 1.45***	0.002	< 0.001	< 0.001
	12 W	7.93 ± 1.12***	9.35 ± 1.28***	7.00 ± 1.07***	< 0.001	< 0.001	< 0.001
Serum 5‐HT, ng/mL	0 W	377.91 ± 39.96	380.26 ± 39.46	379.36 ± 42.32	0.751	0.909	0.851
	2 W	390.42 ± 43.34**	384.16 ± 42.05	408.47 ± 41.38***	0.432	0.002	0.024
	4 W	406.52 ± 42.88***	396.40 ± 47.71**	424.70 ± 40.20***	0.232	0.001	0.020
	8 W	415.11 ± 36.12***	407.72 ± 48.97***	429.95 ± 39.66***	0.359	0.009	0.038
	12 W	420.12 ± 38.18***	408.58 ± 47.39***	436.69 ± 42.35***	0.152	0.001	0.029
Serum E2, pg/mL	0 W	52.11 ± 6.38	53.60 ± 5.43	52.60 ± 5.56	0.177	0.338	0.665
	2 W	53.91 ± 6.81	87.37 ± 6.30***	83.81 ± 5.31***	< 0.001	0.001	< 0.001
	4 W	52.40 ± 5.71	103.55 ± 5.98***	103.10 ± 5.98***	< 0.001	0.694	< 0.001
	8 W	52.35 ± 8.30	109.02 ± 4.95***	112.47 ± 6.13***	< 0.001	0.001	< 0.001
	12 W	52.12 ± 7.60	112.50 ± 5.04***	116.83 ± 5.88***	< 0.001	< 0.001	< 0.001

Serum FSH, mIU/mL	0 W	43.57 ± 4.86	43.49 ± 4.94	43.40 ± 4.62	0.930	0.922	0.849
	2 W	42.71 ± 4.11***	38.04 ± 4.32***	37.91 ± 4.10***	< 0.001	0.875	< 0.001
	4 W	42.93 ± 4.21*	32.36 ± 4.17***	32.27 ± 4.42***	< 0.001	0.914	< 0.001
	8 W	42.66 ± 3.95*	27.31 ± 4.35***	26.84 ± 4.15***	< 0.001	0.565	< 0.001
	12 W	42.02 ± 4.04***	23.66 ± 4.25***	23.14 ± 3.88***	< 0.001	0.507	< 0.001
Serum LH, mIU/mL	0 W	22.90 ± 4.01	22.49 ± 4.18	22.88 ± 3.54	0.596	0.601	0.978
	2 W	22.72 ± 3.32	18.81 ± 3.46***	18.75 ± 3.05***	< 0.001	0.923	< 0.001
	4 W	22.05 ± 3.27**	16.39 ± 3.62***	16.40 ± 2.95***	< 0.001	0.995	< 0.001
	8 W	22.14 ± 3.23*	13.80 ± 3.11***	13.98 ± 2.43***	< 0.001	0.740	< 0.001
	12 W	22.25 ± 2.72	10.40 ± 2.83***	10.32 ± 2.38***	< 0.001	0.873	< 0.001

*Note:* Data are shown as mean ± SD. Using generalized estimation equations (GEEs) analysis. *p*_*a*_ represents the comparison between the Escitalopram group and the Hormone group at the same time point; *p*_*b*_ indicates the comparison of the Hormonal group with the Combined group at the same time point; *p*_*c*_ represents the comparison of the Escitalopram group with the Combined group at the same time point.

Abbreviations: 5‐HT, 5‐hydroxytryptamine; E2, estradiol; ESS, Epworth Sleepiness Scale; FSH, follicle‐stimulating hormone; ISI, Insomnia Severity Index Scale; LH, luteinizing hormone; PSQI, Pittsburgh Sleep Quality Index Scale.

* *p* indicates comparison with the same group before the treatment, *p* < 0.05.

** *p* indicates comparison with the same group before the treatment, *p* < 0.01.

*** *p* indicates comparison with the same group before the treatment, *p* < 0.001.

### 3.3 Changes in Hematologic Indicators

The levels of platelet 5‐HT, platelet 5‐HT7R, and serum 5‐HT in each group are summarized in Table 2 and Table S1. At weeks 2, 4, 8, and 12 after the treatment, the levels of platelet 5‐HT and platelet 5‐HT7R in the three groups were not significantly different from those before the treatment, while the serum 5‐HT levels in the three groups were higher than that before the treatment, with a statistical significance at weeks 4, 8, and 12. Inter‐group comparisons showed a significantly higher serum 5‐HT level in the combined group at weeks 2, 4, 8, and 12 after the treatment than in the other two groups.

The levels of serum E2, FSH, and LH in each group are shown in Table 2. At weeks 2, 4, 8, and 12, serum E2 levels in the Hormone group and Combined group were significantly higher than those before the treatment, while no significant change was evident in the Escitalopram group. At weeks 2, 4, 8, and 12, all three groups reported a decrease in serum FSH levels compared with those before the treatment, with the most significant decrease found in the Combined group.

At weeks 2, 4, 8, and 12, serum LH levels in the three groups were lower than those before the treatment, with significant changes reported in the Hormone group and the Combined group. Inter‐group comparisons reported a significantly lower serum LH level in the Hormone and Combined groups than in the Escitalopram group at weeks 2, 4, 8, and 12, but no significant difference between the Hormone and Combined groups.

### 3.4 Adverse Drug Events

The incidence of AEs during treatment in the three groups is shown in Table S2. There were no serious adverse reactions in any of the three groups. There was no significant difference in the overall incidence of adverse reactions between the different treatment groups. No obvious abnormalities in blood routine, liver and kidney functions, or electrocardiograms were observed in the three groups before and after the treatment.

### 3.5 Correlation Analysis

**FIGURE 3:**
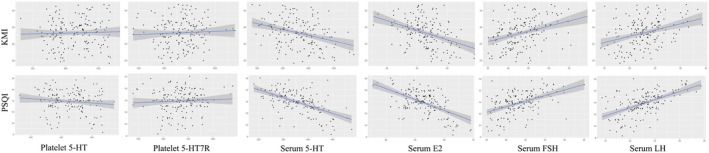
Correlation of peripheral blood indicators with KMI and PSQI scores. Partial Pearson correlation analysis was used after adjustment for age, years of education, and BMI. KMI, Modified Kupperman Menopausal Index; PSQI, Pittsburgh Sleep Quality Index Scale; 5‐HT, 5‐hydroxytryptamine; 5‐HT7R, 5‐hydroxytryptamine 7 receptor; E2, Estradiol; FSH, Follicle‐stimulating Hormone; LH, Luteinizing Hormone.

The correlations between hematological indexes and PSQI, ISI, ESS, and KMI scores are shown in Table S3, Figure 3, and Figure S2. The results showed a moderate negative correlation of serum 5‐HT and serum E2 levels with PSQI score, and a moderate positive correlation of serum FSH and LH levels with PSQI score. Serum E2, serum FSH, and LH levels were weakly correlated with the ISI score. The KMI scores were moderately negatively correlated with serum E2 levels, weakly negatively correlated with serum 5‐HT levels, and weakly positively correlated with serum FSH and LH levels.

## 4 Discussion

In this study, compared with monotherapy, a combination of Femoston and escitalopram reported a better efficacy in treating perimenopausal symptoms and insomnia in perimenopausal women with chronic insomnia, with no significant increase in adverse effects. After 12 weeks of treatment, all three groups showed a decrease in KMI, PSQI, ISI, and ESS scores, as well as in serum FSH levels, along with an increase in serum 5‐HT levels when compared with the baseline levels. Moreover, serum E2 levels increased and serum LH levels decreased in both the Combined and the Hormone groups. Compared with the monotherapy groups, the Combined group showed greater changes in the subjective sleepiness scale score, perimenopausal syndrome score, and hematological indexes. In addition, serum 5‐HT and E2 levels were negatively correlated with PSQI scores, while serum FSH and LH levels were positively correlated with PSQI scores. Serum E2 levels are negatively correlated with KMI scores. These findings suggest that combined therapy may be more effective in treating chronic insomnia in perimenopausal women and that these hematological indices may serve as potential objective indicators of disease severity.

Perimenopausal syndrome is characterized by hot flashes, sweating, mood swings, insomnia, genitourinary symptoms, and other physical symptoms. In our study, KMI scores decreased in the monotherapy groups and the Combined group (Figure S1), with a more pronounced KMI decrease in the Combined group. This finding suggests that Femoston combined with escitalopram has better efficacy in alleviating perimenopausal symptoms in perimenopausal women, consistent with previous findings [23, 26]. Moreover, perimenopausal women are more susceptible to hot flashes and mood disorders, which can exacerbate insomnia [27]. Available studies document that citalopram treatment can simultaneously improve mood and sleep in perimenopausal women [23, 28]. Therefore, we hypothesize that a combined therapy can enhance the effectiveness in alleviating perimenopausal symptoms in perimenopausal women with insomnia by simultaneously improving sleep, mood, and physical symptoms.

In this study, we found a progressive decrease in PSQI, ISI, and ESS scores in all three groups after treatment when compared with the baseline levels (Figure 2). Compared with the monotherapy groups, the Combined group showed the most significant change in subjective sleepiness scale scores at weeks 4 and 12. This may be because the two drugs improve insomnia symptoms in perimenopausal women through different mechanisms. Currently, the mechanism of insomnia in perimenopausal women is believed to be primarily related to decreased E2 levels, frequent vasodilatory symptoms, and changes in the levels of neurotransmitters [6]. Given that the E2 level is correlated with 5‐HT level and that the latter is associated with sleep [9], we speculate that Femoston combined with escitalopram may improve the sleep quality in perimenopausal women with chronic insomnia by increasing their serum E2 and serum 5‐HT levels.

Previous studies have found that perimenopausal women have a low level of E2 and high levels of FSH and LH [13], and the development of insomnia in perimenopausal women is associated with changes in the levels of these sex hormones. Estrogen can affect sleep by modulating thermoregulation, circadian rhythms, vasodilation, oxidative stress, and other physiological processes [6, 29, 30]. Studies have associated decreased estrogen levels to difficulties in sleep initiation and maintenance [7]. Other studies have shown that hormone replacement therapy can increase serum E2 level and decrease FSH and LH levels in perimenopausal women [31, 32]. Consistently, as shown in Figure 2, we found that both the Hormone and Combined groups reported an increased serum E2 level and decreased FSH and LH levels after the treatment when compared with the baseline levels. However, the Escitalopram group reported no significant changes in E2 and LH levels and a slight decrease in FSH after the treatment, in the same line with the finding that escitalopram fails to impact sex hormone levels in the treatment of perimenopausal depression [33]. We hypothesize that escitalopram may improve sleep in perimenopausal women primarily by increasing serum 5‐HT concentrations and improving mood, instead of by altering sex hormone levels.

As mentioned earlier, estrogen levels have been found to correlate with 5‐HT levels. Estrogen can stimulate the synthesis of 5‐HT and inhibit its reuptake by increasing the production of tryptophan hydroxylase and inhibiting the expression of the gene responsible for 5‐HT reuptake [34], thereby increasing the 5‐HT levels. Existing studies evidence that 5‐HT is associated with sleep and that perimenopausal women have lower serum 5‐HT levels [14]. In contrast, hormone replacement therapy can elevate serum 5‐HT levels in postmenopausal women, and SSRI treatment can increase serum 5‐HT levels in perimenopausal women [14, 35]. In this study, we also found that serum 5‐HT levels were elevated in all three groups after the treatments when compared with baseline levels, with more pronounced elevations in the combined group (Figure 2). A potential explanation is that escitalopram oxalate and estrogen may increase the concentration of 5‐HT in peripheral blood through different mechanisms, and that the combined treatment may exert a synergistic effect. Further research is awaited to investigate the specific mechanism. However, one study reports no significant changes in plasma 5‐HT levels in postmenopausal women before and after the hormone therapy [36]. The disparity may be related to the age and menopausal status of the enrolled participants, which needs to be further investigated.

Previous studies have found that 5‐HT7R is associated with sleep and circadian rhythm regulation. Antagonizing 5‐HT7R has been shown to improve rapid eye movement phase sleep in patients with depression, and in animal models, a 5‐HT7R antagonist in combination with escitalopram improved circadian rhythms [37]. In the current study, no significant changes in platelet 5‐HT levels and platelet 5‐HT7R levels were found in the three groups after the treatment. This may be due to the fact that the amount of platelet 5‐HT is easily affected by platelet extraction methods and platelet content, and that the therapeutic effect may be derived from the activity of 5‐HT7R or other pathways, instead of the density changes of 5‐HT7R. Therefore, peripheral platelet 5‐HT and 5‐HT7R levels may not be reliable indicators of efficacy. This speculation needs to be verified in further basic and clinical studies.

As previously mentioned, E2 and 5‐HT levels are associated with perimenopausal symptoms and sleep. Many studies have shown that the serum sex hormone levels in perimenopausal women are correlated with sleep [7, 8]. However, few studies have proven the correlation between 5‐HT and the above symptoms. In the current study, we analyzed the correlation of these hematological indices with KMI scale scores and subjective sleep scale scores. Our study found that serum 5‐HT and E2 levels were negatively correlated with PSQI scores, serum FSH and LH levels were positively correlated with PSQI scores, and serum E2 levels were negatively correlated with KMI scores. Furthermore, we found that sleep improvement in perimenopausal women was accompanied by changes in serum 5‐HT, estrogen, and FSH levels. Therefore, to some extent, peripheral blood serum 5‐HT and serum sex hormone levels can be used as objective indicators to evaluate the severity of chronic insomnia and perimenopausal syndrome in perimenopausal women.

As a randomized controlled study, the strengths of this study are: (1) it evidences that escitalopram combined with Femoston is more effective in treating chronic insomnia in perimenopausal women when compared with the monotherapy; (2) it explored the indicators of the severity of chronic insomnia in the perimenopausal period and illuminates that serum 5‐HT, E2, FSH, and LH levels can serve as objective indicators for the assessment of disease severity in perimenopausal women; and (3) as an RCT study, bias can be minimized to a certain extent, ensuring the objectivity and reliability of the results.

However, there are some limitations in this study: (1) although clinical patients from two centers were collected, the sample size was small and only involved the Chinese population, so it is necessary to expand the sample size and involve more centers and populations to further verify the conclusion; (2) this study lacks a healthy control group; (3) it is unclear how peripheral blood 5‐HT changes in perimenopausal patients without chronic insomnia; (4) the influence of age on 5‐HT changes cannot be ruled out; (5) the follow‐up end point of this study was 12 weeks. The course and long‐term benefits of Femoston combined with escitalopram in the treatment of perimenopausal chronic insomnia remain unclear. For patients on long‐term medications, there is still a lack of specific instructions in the various perimenopausal guidelines on how to reduce the medication dosage or whether to discontinue the medication regimen. Based on our clinical experience, we recommend a one‐year drug regimen maintenance, which can be continued or adjusted according to the patient's clinical symptoms after the one‐year treatment.

In conclusion, compared with hormone or escitalopram alone, a combined therapy of hormone with escitalopram may be more effective in the treatment of perimenopausal chronic insomnia. Serum levels of 5‐HT, E2, FSH, and LH may serve as objective indicators to assess the disease severity in perimenopausal women. These findings can provide insights into clinical treatment.

## Author Contributions

Hui Chen and Shufang Wu conceptualized and coordinated the study, experiment design and performance, data collection and analysis, and manuscript drafting; Hongbin Chen, Guiying Zeng, Weiwei Wu, and Xinyan Chen carried out data collection and analysis. Yingchun Xiao, Ronghua Chen, and Xiujuan Chen carried out data analysis and revised the manuscript. All authors reviewed the results and approved the final manuscript.

## Conflicts of Interest

The authors declare no conflicts of interest.

## Supporting information


**Data S1** Supporting Information

## Data Availability

The data that support the findings of this study are available from the corresponding author. The data are not publicly available due to privacy or ethical restrictions.
