# Supplementary material for: Efficacy and Safety of Hormone Replacement Combined With Escitalopram in the Treatment of Chronic Insomnia in Perimenopausal Women: A Randomized Controlled Trial
Source: CNS Neurosci Ther. 2025 Jun 12;31(6):e70470. doi: 10.1111/cns.70470 (PMC12162946; doi:10.1111/cns.70470)
Supplement: Supplementary file 1 — Data S1 Supporting Information [file CNS-31-e70470-s001.docx]

**ELECTRONIC SUPPLEMENTARY MATERIAL**

**Efficacy and safety of hormone replacement combined with escitalopram in the treatment of chronic insomnia in perimenopausal women: A randomized controlled trial**

**SUPPLEMENTARY METHODS**

**Study design**

*Sample size.* This study was an exploratory trial, with no data reference. Hence, the sample size was not estimated.

*Recruitment.* All interested patients were given a patient information sheet. If they agreed to participate, a signed informed consent was obtained and their medical data were accessed.

*Screening visit.* Participants recruited to the trial underwent structured diagnostic interviews to confirm a diagnosis of chronic insomnia (ICSD-3). Perimenopausal clinical symptoms were assessed by the Modified Kupperman Menopausal Index Scale (KMI) in each group. The Pittsburgh Sleep Quality Index Scale (PSQI), Epworth Sleepiness Scale (ESS), and Insomnia Severity Index Scale (ISI) were used to assess the sleep status of the patients in each group; blood samples were also collected for measurement of full blood count, liver and kidney function panel. Vital signs, height and weight were measured as well.

*Baseline visit.* After the follow-up screening, qualified patients returned for baseline follow-up. They were randomly assigned to the hormone group or the escitalopram group or the combined group and underwent testing of blood monoamine neurotransmitters (platelet 5-HT and 5-HT7R, serum 5-HT, E2, FSH, LH), as well as clinical assessment of the KMI scales and sleepiness scales, including the Insomnia Severity Index Scale (ISI), the Pittsburgh Sleep Quality Index Scale (PSQI), Epworth Sleepiness Scale (ESS).

*Randomization.* Patients were numbered according to the enrollment sequence and randomly grouped at a 1:1:1 allocation ratio according to the order of random numbers generated with SPSS 17.0 software (SPSS Inc., Chicago, IL, USA). An independent researcher used the algorithm to perform rater masking. The randomized information of each eligible patient was sealed in an opaque envelope that corresponded to the patient enrollment number. Participants were randomized into three 12-week treatment regimens: escitalopram + placebo (Escitalopram group), femoston + placebo (Hormone group), and femoston plus escitalopram (Combined group). In the Escitalopram group, escitalopram (Bailot, Sichuan Kelun Pharmaceutical Co., Ltd.) was administered at a dosage of 10 mg once daily; in case of adverse events (AEs) after the administration, the dose was reduced to 5 mg once daily for 3 days and increased to 10 mg for maintenance therapy if the AEs were relieved; the placebo was a femoston analogue and was administered in the same dosage regimen of femoston. In the Hormone group, femoston 1/10 (Solvay Pharmaceutical Co., Ltd.), which consists of a white tablet (estradiol 1mg) and a gray tablet (estradiol 1mg + dydrogesterone 10mg), was given according to the following regimen: 1 tablet orally every day on a 28-day treatment course, with the white tablet taken on the first 14 days and the gray tablet on the next 14 days and a second course starting from day 29. If the discomfort associated with estrogen insufficiency did not improve, the patients received femoston 2/10 (Solvay Pharmaceuticals Ltd.), composed of a brick red tablet (estradiol 2 mg) and a yellow tablet (estradiol 2 mg + dydrogesterone 10 mg), and observed the same dosage scheme. The placebo was an escitalopram analogue, given at a dosage of 10 mg once daily. In the Combined group, femoston and escitalopram were taken simultaneously according to the above schemes. Before treatment, all enrolled patients received a 1-week drug washout period (2 weeks for those already taking a monoamine oxidase inhibitor).

During the treatments, contraindicated drugs were as follows: triptans, antipsychotics, catecholamines, glucocorticosteroids, monoamine oxidase inhibitors and β-receptor blockers. The use of following drugs was allowed: antiplatelet agents, anticoagulants, B vitamins, angiotensin-converting enzyme inhibitors, angiotensin receptor blockers, and calcium channel antagonists.

*Follow-up visit*. Participants were assessed at weeks 2, 4, 8, and 12 after the treatment, within 3 days of the end of the session. Participants underwent a clinical assessment with the same measurements used at baseline follow-up, and blood was taken for analyses of monoamine neurotransmitter and hormone levels.

All assessments were conducted by two neurologists (one senior attending physician and one associate chief physician), who were trained in scale consistency and masked to the grouping and medication of patients.

**Perimenopausal Symptom and Sleep Scale Assessment**

*Modified Kupperman Menopausal Index Scale*: The severity of perimenopausal syndrome was assessed in each group. The KMI consists of 13 items, including hot flashes and sweating (vasodilatory symptoms), sensory abnormalities, insomnia, mood swings, depression, paranoia, dizziness, fatigue, bone and joint pain, headache, palpitations, skin ants, sexual difficulties, and urinary tract infections. Each item has a basic score and a degree score (0-3 points) divided into 4 levels, and the score of each item = basic score × degree score; total score = the sum of the scores of each item. The details of the modified Kupperman scale were as follows: ≤6 points, normal; 7-15 points, mild; 16-30 points, moderate; and >30 points, severe.

*Pittsburgh Sleep Quality Index Scale*: The subjective sleep quality was assessed in each group. The scale consists of seven items, namely subjective sleep quality, sleep latency, sleep duration, sleep efficiency, sleep disorder, use of sleep medication and daytime dysfunction, with each factor scored 0-3 out of 21. A total score of <8 suggested good sleep quality, while that of ≥8 indicated the presence of sleep disorders, with higher scores indicating severer sleep disorders.

*Epworth Sleepiness Scale*: The degree of subjective sleepiness was evaluated in the perimenopausal women. Subjects were asked to assess their likelihood of falling asleep in eight different situations using a scale of 0-3, with a total score of 24. The higher the scores, the greater the severity of symptoms. A score of 7-9 was considered doubtful and that of ≥10 indicated the presence of daytime somnolence, with higher scores indicating greater severity of daytime somnolence.

*Insomnia Severity Index Scale*: The severity of insomnia was evaluated. The scale consists of 7 entries on a 5-point Likert scale, with each entry scored 0-4 and the total score of 0-28, with higher scores indicating severer insomnia. According to the scoring guidelines: 0-7 points, insomnia without significant clinical manifestations; 8-14 points, mild insomnia; 15-21 points, moderate insomnia; and 22-28 points, severe insomnia.

**Blood Sample Detection**

*Collection of blood samples:* All participants had blood samples collected before and at weeks 2, 4, 8 and 12 after the treatment. Three days before blood collection, patients were required not to consume foods that affect the secretion of monoamines and cholinergics, and observe fasting at 22:00 on the evening of the day before blood collection. Blood collection was performed after resting quietly for 30 minutes from 08:00 to 10:00 on the morning of blood collection. Venous blood was collected from the elbow vein into ethylenediaminetetraacetic acid (EDTA) anticoagulant tubes (1 tube) and procoagulant tubes (2 tubes), followed by platelet and serum extraction.

*Sample handling and detection:* Platelets were extracted with a human peripheral platelet separation kit (Solarbio, Beijing, China), and serum was extracted by a low-speed benchtop centrifuge (Shanghai Anting Scientific Instrument Factory, China, No. type: TDL-50B). Platelets and serum were frozen at -80 °C to avoid repeated freeze-thaw, and the storage time was not more than 2 months. Enzyme-linked immunosorbent assay (ELISA) was adopted to measure platelet 5-HT, platelet 5-HT7R, serum E2, FSH, LH and 5-HT levels.

**SUPPLEMENTARY TABLES**

**Table S1. Changes in KMI scores, platelet 5-HT and 5-HT7R levels in the three groups before and after the treatment**

| Item | Time | | Escitalopram group (n=58) | | Hormone group  (n=55) | | Combined group (n=53) | *P_a_* | *P_b_* | *P_c_* |
| --- | --- | --- | --- | --- | --- | --- | --- | --- | --- | --- |
| KMI Score | 0W | | 28.14±4.18 | | 28.04±4.24 | | 28.64±4.16 | 0.897 | 0.450 | 0.521 |
|  | 2W | | 24.33±3.55^***^ | | 25.18±3.49^***^ | | 23.13±2.77^***^ | 0.193 | 0.001 | 0.045 |
|  | 4W | | 18.34±1.97^***^ | | 20.11±1.65^***^ | | 16.42±1.98^***^ | ＜0.001 | ＜0.001 | ＜0.001 |
|  | 8W | | 17.00±2.09^***^ | | 19.09±2.30^***^ | | 13.92±1.73^***^ | ＜0.001 | ＜0.001 | ＜0.001 |
|  | 12W | | 14.95±1.69^***^ | | 16.64±2.17^***^ | | 13.49±1.66^***^ | ＜0.001 | ＜0.001 | ＜0.001 |
| Platelet 5-HT,  ng/ml | 0W | | 428.74±53.50 | | 422.11±68.45 | | 426.74±60.04 | All ＞0.05^#^ | | |
|  | 2W | | 425.65±62.23 | | 430.18±65.94 | | 429.69±65.06 |  |  |  |
|  | 4W | | 420.00±61.81 | | 422.48±65.22 | | 416.16±57.53 |  |  |  |
|  | 8W | | 423.84±65.32 | | 427.85±66.50 | | 428.67±56.30 |  |  |  |
|  | 12W | | 437.63±61.02 | | 419.94±64.09 | | 435.40±55.12 |  |  |  |
| Platelet 5-HT7R, ng/l | | 0W | | 671.53±75.08 | | 666.28±77.45 | 660.82±60.97 | All ＞0.05^#^ | | |
|  |  | 2W | | 669.16±61.75 | | 661.59±59.66 | 657.28±54.97 |  |  |  |
|  |  | 4W | | 663.41±57.68 | | 664.67±45.16 | 668.79±46.46 |  |  |  |
|  |  | 8W | | 662.33±69.34 | | 661.56±40.27 | 666.89±45.91 |  |  |  |
|  |  | 12W | | 673.84±55.00 | | 677.86±62.65 | 674.20±41.74 |  |  |  |

Data are shown as mean ± standard deviation. KMI, Modified kupperman menopausal index; 5-HT, 5-hydroxytryptamine; 5-HT7R, 5-hydroxytryptamine 7 receptor; Using generalized estimation equations (GEEs) analysis.

*P_a_* represents the comparison between the Escitalopram group and the Hormone group at the same time point; *P_b_* indicates the comparison of the Hormonal group with the Combined group at the same time point; *P_c_* represents the comparison of the Escitalopram group with the Combined group at the same time point. ^#^*P* represents a comparison between three groups at the same point in time. ^*^*P* indicates comparison with the same group before the treatment, *P*＜0.05；^**^*P* indicates comparison with the same group before the treatment, *P*＜0.01；^***^*P* indicates comparison with the same group before the treatment, *P*＜0.001.

**Table S2. Comparison of adverse event rates**

| **Time** | **Escitalopram group (n=58)** | **Hormone group**  **(n=55)** | **Combined group (n=53)** | **χ² value** | **^#^*P value*** |
| --- | --- | --- | --- | --- | --- |
| 0-2W | 5（8.8%） | 5（9.1%） | 5（9.4%） | - | - |
| 3-4W | 3（5.2%） | 3（5.4%） | 4（7.5%） | - | - |
| 5-8W | 0 （0%） | 0（0%） | 0（0%） | - | - |
| 9-12W | 0（0%） | 0（0%） | 0（0%） | - | - |
| Total | 13.8% | 14.5% | 17.0% | 0.237 | 0.888 |

Data are shown as n (%).

^#^*P* value represents a comparison between three groups at the same time point.

**Table S3. Partial Pearson correlation coefficient (r) between clinical efficacy and peripheral blood indices^a^.**

| **Item** | **PSQI** | |  | **ISI** | |  | **ESS** | |  | **KMI** | |
| --- | --- | --- | --- | --- | --- | --- | --- | --- | --- | --- | --- |
|  | *r* | *P* value |  | *r* | *P* value |  | *r* | *P* value |  | *r* | *P* value |
| Platelet 5-HT | -0.119 | 0.131 |  | -0.011 | 0.893 |  | -0.015 | 0.850 |  | 0.035 | 0.653 |
| Serum 5-HT | -0.560 | ＜0.001 |  | -0.136 | 0.083 |  | -0.019 | 0.808 |  | -0.340 | ＜0.001 |
| Serum E2 | -0.557 | ＜0.001 |  | -0.233 | 0.003 |  | 0.074 | 0.350 |  | -0.402 | ＜0.001 |
| Serum FSH | 0.468 | ＜0.001 |  | 0.212 | 0.007 |  | 0.042 | 0.592 |  | 0.354 | ＜0.001 |
| Serum LH | 0.549 | ＜0.001 |  | 0.177 | 0.023 |  | -0.056 | 0.477 |  | 0.353 | ＜0.001 |
| Platelet 5-HT7R | 0.062 | 0.435 |  | 0.023 | 0.771 |  | 0.128 | 0.102 |  | 0.055 | 0.489 |

Data are shown as correlation coefficients. 5-HT, 5-hydroxytryptamine; 5-HT7R, 5-hydroxytryptamine 7 receptor; E2, estradiol; FSH, follicle-stimulating hormone; LH, luteinizing hormone.

^a^ Partial correlation analyses were adjusted for age, years of education, and BMI level.

**
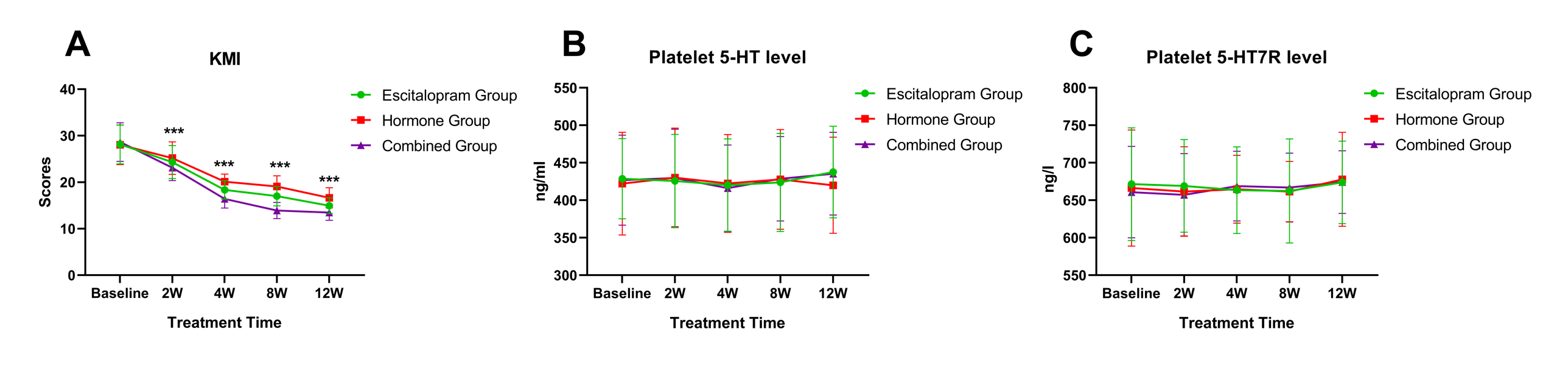
**

**Fig. S1 Changes in KMI scale scores and platelet 5-HT and 5-HT7R levels at different time points in the three groups.** KMI, Modified kupperman menopausal index; 5-HT, 5-hydroxytryptamine; 5-HT7R, 5-hydroxytryptamine 7 receptor; Comparison of three groups at the same time point:*^*^ P*<0.05，*^**^ P*＜0.01，*^***^ P*＜0.001.

**
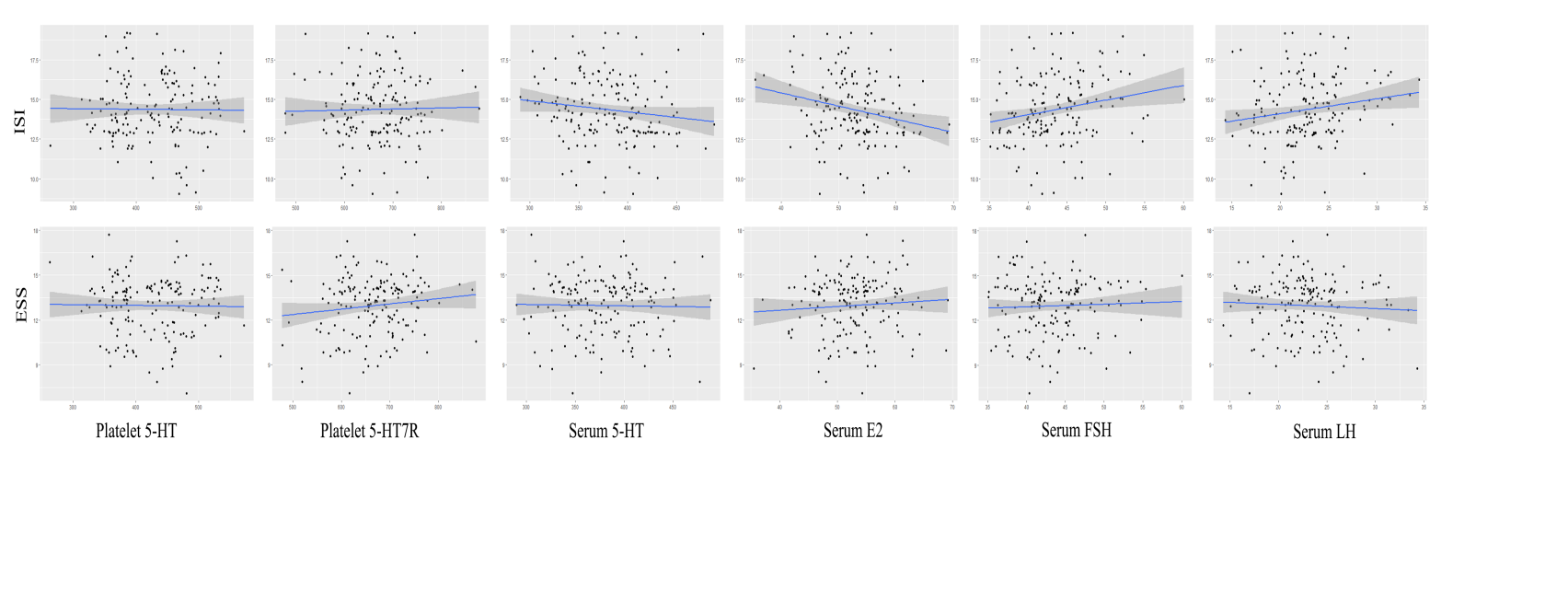
**

**Fig.S2 Correlation of peripheral blood indicators with ISI and ESS scores.** Partial Pearson correlation analysis was used after adjustment for age, years of education, and BMI. ISI, Insomnia Severity Index Scale; ESS, Epworth Sleepiness Scale; 5-HT, 5-hydroxytryptamine; 5-HT7R, 5-hydroxytryptamine 7 receptorE2, Estradiol; FSH, Follicle-stimulating Hormone; LH, Luteinizing Hormone.
